# (1*R*,2*R*)-2-(Pyridin-4-yl­methyl­amino)­cyclo­hexa­naminium chloride

**DOI:** 10.1107/S1600536811005526

**Published:** 2011-02-19

**Authors:** Lin Cheng, Li-Min Zhang, Jian-Quan Wang

**Affiliations:** a Department of Chemistry and Chemical Engineering, Southeast University, Nanjing 211189, People’s Republic of China

## Abstract

In the crystal structure of the title compound, C_12_H_20_N_3_^+^·Cl^−^, the protonated (1*R*,2*R*)-(pyridin-4-ylmeth­yl)cyclo­hexane-1,2-diamine cations and chloride anions are linked *via* N—H⋯N and N—H⋯Cl hydrogen bonds into a three-dimensional network.

## Related literature

For coordination polymers, see: He *et al.* (2010 ▶). For related structures, see: Gou *et al.* (2010 ▶).
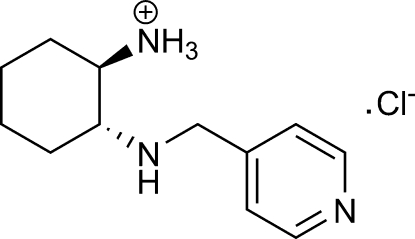


## Experimental

### 

#### Crystal data

C_12_H_20_N_3_^+^·Cl^−^
*M* *_r_* = 241.76
Orthorhombic, 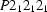

*a* = 5.5256 (10) Å
*b* = 13.928 (2) Å
*c* = 16.685 (3) Å
*V* = 1284.1 (4) Å^3^
*Z* = 4
Mo *K*α radiation
μ = 0.28 mm^−1^
*T* = 291 K
0.25 × 0.20 × 0.18 mm

#### Data collection

Bruker SMART APEX CCD diffractometer
Absorption correction: multi-scan (*SADABS*; Sheldrick, 1995 ▶) *T*_min_ = 0.934, *T*_max_ = 0.952
5296 measured reflections
2516 independent reflections
2259 reflections with *I* > 2σ(*I*)
*R*_int_ = 0.019

#### Refinement

*R*[*F* ^2^ > 2σ(*F* ^2^)] = 0.041
*wR*(*F* ^2^) = 0.102
*S* = 1.06
2516 reflections
145 parameters
H-atom parameters constrained
Δρ_max_ = 0.26 e Å^−3^
Δρ_min_ = −0.24 e Å^−3^
Absolute structure: Flack (1983 ▶), 1031 Friedel pairs
Flack parameter: −0.04 (8)

### 

Data collection: *SMART* (Bruker, 2000 ▶); cell refinement: *SAINT-Plus* (Bruker, 2000 ▶); data reduction: *SAINT-Plus*; program(s) used to solve structure: *SHELXS97* (Sheldrick, 2008 ▶); program(s) used to refine structure: *SHELXL97* (Sheldrick, 2008 ▶); molecular graphics: *ORTEPIII* (Burnett & Johnson, 1996 ▶) and *PLATON* (Spek, 2009 ▶); software used to prepare material for publication: *SHELXTL* (Sheldrick, 2008 ▶).

## Supplementary Material

Crystal structure: contains datablocks I, global. DOI: 10.1107/S1600536811005526/bt5470sup1.cif

Structure factors: contains datablocks I. DOI: 10.1107/S1600536811005526/bt5470Isup2.hkl

Additional supplementary materials:  crystallographic information; 3D view; checkCIF report

## Figures and Tables

**Table 1:** Hydrogen-bond geometry (Å, °)

*D*—H⋯*A*	*D*—H	H⋯*A*	*D*⋯*A*	*D*—H⋯*A*
N1—H1*B*⋯N3^i^	0.89	2.13	2.926 (2)	148
N1—H1*C*⋯Cl1	0.89	2.32	3.201 (2)	172
N1—H1*D*⋯Cl1^ii^	0.89	2.28	3.1583 (19)	170
N2—H2*C*⋯Cl1^iii^	0.89	2.72	3.5538 (19)	157

Symmetry codes: (i) 
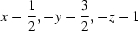
; (ii) 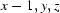; (iii) 
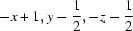
.

**Fig. 1.**
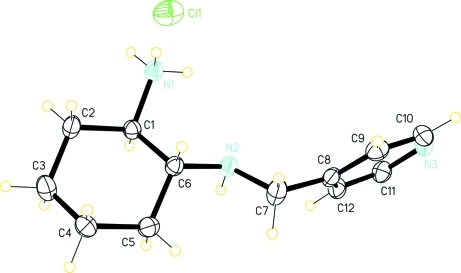
View of the asymmetric unit of the title compoundcompound with the atom-labelling scheme. Displacement ellipsoids are drawn at the 30% probability level. H atoms are represented as small spheres of arbitrary radii.

**Fig. 2.**
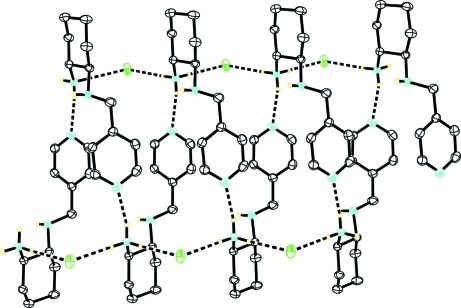
View of the one-dimesional hydrogen bonding chain along the a axis.

**Fig. 3.**
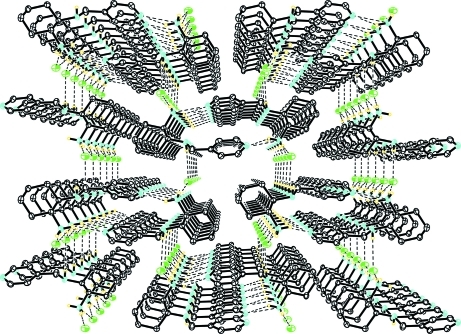
View of the three-dimensional supramolecular network along the bc plane.

## supplementary crystallographic information

### Comment

Recent years have witnessed an explosion of great interest in chiral coordination polymers because of their potential utility in enantiomerically selective catalysis and separations, second-order nonlinearoptical (NLO) applications and magnetism (He *et al.* 2010). We tried to synthesize such polymers by use of chiral (1*R*,2*R*)-(pyridin-4-ylmethyl)cyclohexane-1,2-diamine ligand and zinc chloride. However, Zn(II) ions weren't ligated to the chiral ligands and the hydrochloride of the ligand has been obtained in the reaction conditions. Herein, we report the structure of this hydrochloride, 1.HCl [1 = (1*R*,2*R*)-(pyridin-4-ylmethyl)cyclohexane-1,2-diamine].

The asymmetric unit of the title compound contains a protonated (1*R*,2*R*)-(pyridin-4-ylmethyl)cyclohexane-1,2-diamine and a chloride ion. In the molecule, the distances of the C—N bonds of the pyridine ring are 1.331 (3) and 1.338 (3) Å, which are shorter than those of C—N bonds (1.452 (3), 1.478 (2) and 1.498 (2) Å) of cyclohexane-1,2-diamine. The protonated (1*R*,2*R*)-(pyridin-4-ylmethyl)cyclohexane-1,2-diamine cations and chloride anions are linked to each other, *via* N—H···N (N1···N3a 2.926 (2) Å, symmetry code: a, -1/2 + *x*, -3/2 - *y*, -1 - *z*) and N—H···Cl (N1···Cl1 3.201 (2) Å, N1···Cl1b 3.158 (2) Å, symmetry code: b, -1 + *x*, *y*, *z*) hydrogen bonds between the N atoms of aminium and the N atoms of adjacent pyridine rings, as well as the N atoms of aminium and chloride anions into a one-dimensional hydrogen bonding chain along the *a* axis (Fig.2), which are further constructed into a three-dimensional supramolecular network by interchain N—H···Cl hydrogen-bonds (N2···Cl1c 3.554 (2) Å, symmetry code: c, 1 - *x*, -1/2 + *y*, -1/2 - *z*) between secondary amines and chloride anions.

### Experimental

1*R*,2*R*)-(pyridin-4-ylmethyl)cyclohexane-1,2-diamine (0.021 g, 0.1 mmol) dissolved in water (5 ml) was added to a methanol solution (10 ml) ZnCl_2_ (0.019 g, 0.1 mmol). The mixture solution was stirred for 2 h at room temperature and then filtered. The filtrate was allowed to evaporate slowly at room temperature. After 2 weeks, colorless block crystals were obtained in 33.1% yield (0.008 g).

### Refinement

All H atoms attached to C atoms were fixed geometrically and treated as riding with C—H = 0.93–0.97 Å with *U*_iso_(H) = 1.2 *U*_eq_(C). H atoms attached to N atoms were located in difference Fourier maps and included in the subsequent refinement using restraints (N—H= 0.89 (1) Å) with *U*_iso_(H) = 1.5 *U*_eq_(N).

### Crystal data

**Table:**

C_12_H_20_N_3_^+^·Cl^−^	*F*(000) = 520
*M**_r_* = 241.76	*D*_x_ = 1.251 Mg m^−^^3^
Orthorhombic, *P*2_1_2_1_2_1_	Mo *K*α radiation, λ = 0.71073 Å
Hall symbol: P 2ac 2ab	Cell parameters from 780 reflections
*a* = 5.5256 (10) Å	θ = 2.5–28.0°
*b* = 13.928 (2) Å	µ = 0.28 mm^−^^1^
*c* = 16.685 (3) Å	*T* = 291 K
*V* = 1284.1 (4) Å^3^	Block, colorless
*Z* = 4	0.25 × 0.20 × 0.18 mm

### Data collection

**Table:**

Bruker SMART APEX CCD diffractometer	2516 independent reflections
Radiation source: fine-focus sealed tube	2259 reflections with *I* > 2σ(*I*)
graphite	*R*_int_ = 0.019
φ and ω scans	θ_max_ = 26.0°, θ_min_ = 1.9°
Absorption correction: multi-scan (*SADABS*; Sheldrick, 1995)	*h* = −6→6
*T*_min_ = 0.934, *T*_max_ = 0.952	*k* = −17→16
5296 measured reflections	*l* = −6→20

### Refinement

**Table:**

Refinement on *F*^2^	Secondary atom site location: difference Fourier map
Least-squares matrix: full	Hydrogen site location: inferred from neighbouring sites
*R*[*F*^2^ > 2σ(*F*^2^)] = 0.041	H-atom parameters constrained
*wR*(*F*^2^) = 0.102	*w* = 1/[σ^2^(*F*_o_^2^) + (0.0552*P*)^2^ + 0.1405*P*] where *P* = (*F*_o_^2^ + 2*F*_c_^2^)/3
*S* = 1.06	(Δ/σ)_max_ < 0.001
2516 reflections	Δρ_max_ = 0.26 e Å^−^^3^
145 parameters	Δρ_min_ = −0.24 e Å^−^^3^
0 restraints	Absolute structure: Flack (1983), 1031 Friedel pairs
Primary atom site location: structure-invariant direct methods	Flack parameter: −0.04 (8)

### Special details

**Table:**

Geometry. All e.s.d.'s (except the e.s.d. in the dihedral angle between two l.s. planes) are estimated using the full covariance matrix. The cell e.s.d.'s are taken into account individually in the estimation of e.s.d.'s in distances, angles and torsion angles; correlations between e.s.d.'s in cell parameters are only used when they are defined by crystal symmetry. An approximate (isotropic) treatment of cell e.s.d.'s is used for estimating e.s.d.'s involving l.s. planes.
Refinement. Refinement of *F*^2^ against ALL reflections. The weighted *R*-factor *wR* and goodness of fit *S* are based on *F*^2^, conventional *R*-factors *R* are based on *F*, with *F* set to zero for negative *F*^2^. The threshold expression of *F*^2^ > σ(*F*^2^) is used only for calculating *R*-factors(gt) *etc*. and is not relevant to the choice of reflections for refinement. *R*-factors based on *F*^2^ are statistically about twice as large as those based on *F*, and *R*- factors based on ALL data will be even larger.

### Fractional atomic coordinates and isotropic or equivalent isotropic displacement parameters (Å^2^)

**Table:**

	*x*	*y*	*z*	*U*_iso_*/*U*_eq_	
Cl1	0.80278 (10)	−0.57398 (4)	−0.18879 (4)	0.05595 (19)	
C1	0.2910 (4)	−0.76369 (13)	−0.19230 (10)	0.0334 (4)	
H1A	0.1413	−0.7975	−0.2063	0.040*	
C2	0.2862 (4)	−0.74096 (15)	−0.10296 (10)	0.0411 (5)	
H2A	0.4249	−0.7015	−0.0894	0.049*	
H2B	0.1410	−0.7048	−0.0905	0.049*	
C3	0.2912 (5)	−0.83212 (16)	−0.05335 (12)	0.0500 (6)	
H3A	0.2945	−0.8158	0.0032	0.060*	
H3B	0.1459	−0.8692	−0.0636	0.060*	
C4	0.5108 (5)	−0.89136 (16)	−0.07397 (12)	0.0489 (6)	
H4A	0.5090	−0.9502	−0.0429	0.059*	
H4B	0.6559	−0.8560	−0.0599	0.059*	
C5	0.5163 (5)	−0.91576 (15)	−0.16282 (12)	0.0464 (5)	
H5A	0.3803	−0.9571	−0.1754	0.056*	
H5B	0.6636	−0.9509	−0.1746	0.056*	
C6	0.5051 (4)	−0.82635 (13)	−0.21600 (10)	0.0341 (4)	
H6A	0.6531	−0.7892	−0.2067	0.041*	
C7	0.7097 (5)	−0.89100 (19)	−0.33387 (12)	0.0534 (6)	
H7A	0.7163	−0.9575	−0.3168	0.064*	
H7B	0.8493	−0.8583	−0.3116	0.064*	
C8	0.7219 (4)	−0.88678 (13)	−0.42405 (11)	0.0371 (5)	
C9	0.9147 (4)	−0.84386 (15)	−0.46266 (14)	0.0439 (5)	
H9A	1.0437	−0.8198	−0.4330	0.053*	
C10	0.9160 (5)	−0.83668 (16)	−0.54485 (14)	0.0476 (6)	
H10A	1.0495	−0.8084	−0.5693	0.057*	
C11	0.5538 (4)	−0.91212 (16)	−0.55406 (12)	0.0447 (5)	
H11A	0.4288	−0.9367	−0.5852	0.054*	
C12	0.5397 (4)	−0.92329 (16)	−0.47230 (12)	0.0432 (5)	
H12A	0.4087	−0.9551	−0.4495	0.052*	
N1	0.2983 (3)	−0.67078 (11)	−0.23746 (9)	0.0377 (4)	
H1B	0.3011	−0.6827	−0.2899	0.057*	
H1C	0.4305	−0.6382	−0.2237	0.057*	
H1D	0.1676	−0.6362	−0.2256	0.057*	
N2	0.4907 (3)	−0.84717 (12)	−0.30272 (9)	0.0380 (4)	
H2C	0.3781	−0.8927	−0.3056	0.057*	
N3	0.7369 (4)	−0.86795 (12)	−0.59140 (10)	0.0447 (5)	

### Atomic displacement parameters (Å^2^)

**Table:**

	*U*^11^	*U*^22^	*U*^33^	*U*^12^	*U*^13^	*U*^23^
Cl1	0.0377 (3)	0.0491 (3)	0.0811 (4)	0.0058 (3)	−0.0025 (3)	0.0005 (3)
C1	0.0329 (9)	0.0365 (9)	0.0309 (9)	0.0019 (8)	0.0000 (9)	0.0007 (7)
C2	0.0437 (12)	0.0489 (11)	0.0308 (9)	0.0119 (11)	0.0016 (9)	−0.0052 (8)
C3	0.0536 (13)	0.0626 (14)	0.0340 (10)	0.0020 (13)	0.0029 (10)	0.0052 (10)
C4	0.0653 (16)	0.0475 (12)	0.0340 (10)	0.0102 (12)	−0.0032 (11)	0.0073 (9)
C5	0.0638 (15)	0.0375 (11)	0.0379 (10)	0.0082 (12)	−0.0023 (10)	−0.0008 (9)
C6	0.0363 (11)	0.0359 (10)	0.0301 (9)	0.0041 (9)	−0.0018 (8)	−0.0026 (7)
C7	0.0519 (13)	0.0710 (15)	0.0372 (10)	0.0208 (13)	0.0009 (10)	−0.0047 (10)
C8	0.0406 (12)	0.0354 (10)	0.0353 (9)	0.0088 (9)	0.0034 (9)	−0.0056 (8)
C9	0.0408 (12)	0.0395 (11)	0.0514 (12)	−0.0010 (10)	−0.0041 (10)	−0.0065 (10)
C10	0.0457 (13)	0.0437 (12)	0.0532 (13)	−0.0009 (10)	0.0117 (11)	0.0075 (10)
C11	0.0435 (12)	0.0491 (12)	0.0414 (11)	−0.0011 (11)	−0.0022 (9)	−0.0093 (10)
C12	0.0382 (11)	0.0475 (12)	0.0439 (11)	−0.0031 (11)	0.0072 (9)	−0.0033 (10)
N1	0.0384 (9)	0.0403 (9)	0.0344 (8)	0.0069 (8)	−0.0010 (7)	−0.0009 (7)
N2	0.0415 (10)	0.0421 (9)	0.0304 (8)	0.0065 (8)	0.0013 (8)	−0.0049 (7)
N3	0.0506 (12)	0.0458 (10)	0.0377 (8)	0.0048 (9)	0.0064 (8)	−0.0002 (7)

### Geometric parameters (Å, °)

**Table:**

C1—N1	1.498 (2)	C7—N2	1.452 (3)
C1—C6	1.523 (3)	C7—C8	1.507 (3)
C1—C2	1.524 (2)	C7—H7A	0.9700
C1—H1A	0.9800	C7—H7B	0.9700
C2—C3	1.516 (3)	C8—C9	1.381 (3)
C2—H2A	0.9700	C8—C12	1.386 (3)
C2—H2B	0.9700	C9—C10	1.375 (3)
C3—C4	1.507 (3)	C9—H9A	0.9300
C3—H3A	0.9700	C10—N3	1.331 (3)
C3—H3B	0.9700	C10—H10A	0.9300
C4—C5	1.521 (3)	C11—N3	1.338 (3)
C4—H4A	0.9700	C11—C12	1.375 (3)
C4—H4B	0.9700	C11—H11A	0.9300
C5—C6	1.530 (3)	C12—H12A	0.9300
C5—H5A	0.9700	N1—H1B	0.8900
C5—H5B	0.9700	N1—H1C	0.8900
C6—N2	1.478 (2)	N1—H1D	0.8900
C6—H6A	0.9800	N2—H2C	0.8899
			
N1—C1—C6	110.08 (15)	C1—C6—H6A	107.7
N1—C1—C2	108.24 (14)	C5—C6—H6A	107.7
C6—C1—C2	112.74 (16)	N2—C7—C8	112.22 (19)
N1—C1—H1A	108.6	N2—C7—H7A	109.2
C6—C1—H1A	108.6	C8—C7—H7A	109.2
C2—C1—H1A	108.6	N2—C7—H7B	109.2
C3—C2—C1	111.09 (16)	C8—C7—H7B	109.2
C3—C2—H2A	109.4	H7A—C7—H7B	107.9
C1—C2—H2A	109.4	C9—C8—C12	116.63 (18)
C3—C2—H2B	109.4	C9—C8—C7	121.1 (2)
C1—C2—H2B	109.4	C12—C8—C7	122.2 (2)
H2A—C2—H2B	108.0	C10—C9—C8	120.1 (2)
C4—C3—C2	110.41 (19)	C10—C9—H9A	120.0
C4—C3—H3A	109.6	C8—C9—H9A	120.0
C2—C3—H3A	109.6	N3—C10—C9	123.6 (2)
C4—C3—H3B	109.6	N3—C10—H10A	118.2
C2—C3—H3B	109.6	C9—C10—H10A	118.2
H3A—C3—H3B	108.1	N3—C11—C12	123.8 (2)
C3—C4—C5	111.15 (19)	N3—C11—H11A	118.1
C3—C4—H4A	109.4	C12—C11—H11A	118.1
C5—C4—H4A	109.4	C11—C12—C8	119.6 (2)
C3—C4—H4B	109.4	C11—C12—H12A	120.2
C5—C4—H4B	109.4	C8—C12—H12A	120.2
H4A—C4—H4B	108.0	C1—N1—H1B	109.5
C4—C5—C6	112.48 (16)	C1—N1—H1C	109.5
C4—C5—H5A	109.1	H1B—N1—H1C	109.5
C6—C5—H5A	109.1	C1—N1—H1D	109.5
C4—C5—H5B	109.1	H1B—N1—H1D	109.5
C6—C5—H5B	109.1	H1C—N1—H1D	109.5
H5A—C5—H5B	107.8	C7—N2—C6	112.84 (16)
N2—C6—C1	108.96 (15)	C7—N2—H2C	105.3
N2—C6—C5	114.22 (15)	C6—N2—H2C	103.3
C1—C6—C5	110.31 (16)	C10—N3—C11	116.16 (17)
N2—C6—H6A	107.7		

### Hydrogen-bond geometry (Å, °)

**Table:**

*D*—H···*A*	*D*—H	H···*A*	*D*···*A*	*D*—H···*A*
N1—H1B···N3^i^	0.89	2.13	2.926 (2)	148
N1—H1C···Cl1	0.89	2.32	3.201 (2)	172
N1—H1D···Cl1^ii^	0.89	2.28	3.1583 (19)	170
N2—H2C···Cl1^iii^	0.89	2.72	3.5538 (19)	157

Symmetry codes: (i) *x*−1/2, −*y*−3/2, −*z*−1; (ii) *x*−1, *y*, *z*; (iii) −*x*+1, *y*−1/2, −*z*−1/2.
